# Reproductive Endocrine Stability Despite Persistent Hypogonadism in Well-Chelated Adult Women with Transfusion-Dependent β-Thalassemia

**DOI:** 10.3390/jcm15041418

**Published:** 2026-02-11

**Authors:** Ergul Demircivi, Melis Altug Inan, Nurgul Bulut, Fadime Ersoy Dursun, Abdulkadir Turgut

**Affiliations:** 1 Department of Obstetrics and Gynaecology, Istanbul Medeniyet University, Göztepe Prof. Dr. Süleyman Yalçın Training and Research Hospital, 34722 Istanbul, Türkiye; 2 Department of Biostatistics and Medical Informatics, Faculty of Medicine, Istanbul Medeniyet University, Göztepe Prof. Dr. Süleyman Yalçın Training and Research Hospital, 34722 Istanbul, Türkiye; 3 Department of Haematology, Istanbul Medeniyet University, Göztepe Prof. Dr. Süleyman Yalçın Training and Research Hospital, 34722 Istanbul, Türkiye

**Keywords:** transfusion-dependent β-thalassemia, hypogonadism, reproductive endocrinology, anti-Müllerian hormone, endocrine complications

## Abstract

**Background:** Endocrine complications remain a major cause of long-term morbidity in patients with transfusion-dependent β-thalassemia (TDT), with hypogonadism being the most frequently reported abnormality. Although iron overload is central to disease pathophysiology, its relationship with reproductive endocrine function in well-chelated adult women remains unclear. **Methods:** This retrospective longitudinal study evaluated endocrine function in 15 adult women with transfusion-dependent β-thalassemia major over a two-year follow-up period at a tertiary care center. Age, hormonal profiles, ovarian reserve markers, and clinical reproductive characteristics were assessed at baseline and follow-up. An age-matched control group of 22 healthy women was included. Endocrine and biochemical evaluation comprised gonadotropins (follicle-stimulating hormone and luteinizing hormone), estradiol, thyroid-stimulating hormone, prolactin, anti-Müllerian hormone, hemoglobin, serum iron, total iron-binding capacity, vitamin B12, folate, 25-hydroxyvitamin D, and cardiac and hepatic MRI T2* assessment of iron burden. **Results:** Hypogonadism was clinically prevalent, while other endocrine axes largely remained within reference ranges during follow-up. No newly emerging overt endocrine disorders were identified. Reproductive hormone levels showed no significant temporal changes and were comparable to those of healthy controls. AMH levels demonstrated marked interindividual variability and did not consistently correlate with systemic or imaging-based iron indices. **Conclusions:** In well-chelated adult women with transfusion-dependent β-thalassemia, reproductive endocrine parameters appear biochemically stable over short-term follow-up, yet clinically relevant hypogonadism persists. AMH variability may reflect subtle ovarian reserve impairment not captured by conventional gonadotropin measurements, supporting the need for longitudinal, phenotype-oriented endocrine surveillance.

## 1. Introduction

Beta-thalassemia major (β-TM) is one of the most common severe monogenic hemoglobinopathies worldwide, largely due to the heterozygote advantage against malaria [1,2]. This hereditary condition is particularly prevalent in Mediterranean, Middle Eastern, and Southeast Asian populations. The disorder results from reduced or absent β-globin chain synthesis, leading to ineffective erythropoiesis, chronic hemolysis, and severe anemia [1,3]. Patients with marked β-globin deficiency require lifelong regular transfusions and are therefore classified as having transfusion-dependent β-thalassemia (TDT) [4]. Although advances in transfusion strategies and modern iron chelation therapies have markedly improved survival, transfusional iron overload remains the major determinant of long-term morbidity [1].

Progressive iron accumulation resulting from lifelong transfusions and increased intestinal iron absorption leads to widespread organ toxicity, with the endocrine system being among the most frequently affected targets [1,5]. Consequently, iron overload–related endocrinopathies constitute a substantial component of long-term morbidity in TDT. Dysfunction of the hypothalamic–pituitary–gonadal (HPG) axis represents a major endocrine complication, and hypogonadism is consistently reported as the most frequent hormonal abnormality. Iron deposition within the hypothalamus and anterior pituitary occurs early in the disease course and may remain clinically silent for prolonged periods before the development of overt endocrine dysfunction [6]. Gonadotroph cells are particularly vulnerable to iron-induced oxidative damage, resulting in impaired gonadotropin secretion and clinical consequences such as delayed or arrested puberty, menstrual disturbances, infertility, and reduced bone mineral density. Although ovarian iron deposition may also contribute, the predominant mechanism appears to involve central hypogonadism, as supported by the frequent discordance observed between gonadal dysfunction and contemporaneous systemic iron overload indices [1,6]. Higher serum ferritin levels, lower pre-transfusion hemoglobin concentrations, and chronic hypoxia have consistently been associated with an increased risk of pubertal impairment and gonadal dysfunction. In women with β-TM, reproductive concerns extend beyond hypogonadism to include the preservation of ovarian reserve [1,7].

Anti-Müllerian hormone (AMH), a relatively stable marker of the growing follicle pool, has been widely studied in this context; however, available data remain conflicting. While several studies suggest that ovarian reserve may be relatively preserved despite HPG axis dysfunction, others indicate that chronic hemosiderosis and oxidative stress may contribute to follicular depletion [1]. These discrepancies underscore the need for focused investigations specifically addressing reproductive endocrinology in female patients with TDT.

Iron overload also affects multiple other endocrine axes, contributing to thyroid dysfunction, impaired glucose metabolism, adrenal axis abnormalities, and reduced bone mineral density. Iron deposition in pancreatic β-cells may impair insulin secretion, whereas accumulation within the thyroid gland or the pituitary–adrenal axis may result in subclinical or overt hormonal disturbances. Although iron chelation therapy is essential for reducing iron burden, certain chelating agents have been associated with alterations in bone turnover and gonadal responsiveness. Despite improvements in contemporary chelation regimens, endocrine dysfunction may be only partially reversible, reflecting the cumulative and sometimes irreversible nature of iron-induced endocrine injury [5,8].

Although endocrine complications in β-thalassemia major have been widely investigated, studies focusing specifically on adult female patients with transfusion-dependent disease remain limited and often heterogeneous. Given the impact of hypogonadism, menstrual disturbances, fertility potential, thyroid dysfunction, adrenal insufficiency, and glucose dysregulation on long-term health and quality of life, a comprehensive evaluation of endocrine parameters in well-defined adult female cohorts is warranted.

In this context, the present study evaluates endocrine function in adult women with TDT over a two-year follow-up period, with particular emphasis on reproductive endocrine parameters, ovarian reserve, and their relationship with biochemical and imaging-based indices of iron overload. By integrating hormonal profiles with clinical reproductive characteristics, this study aims to provide insight into the mechanisms underlying endocrine dysfunction in adulthood and to clarify the limited concordance between gonadal impairment and current systemic iron burden.

## 2. Materials and Methods

### 2.1. Study Design and Participants

This study was designed as a retrospective, descriptive, and comparative longitudinal analysis including adult women (≥18 years) with a confirmed diagnosis of TDT who had available endocrine measurements at baseline and after a two-year follow-up period. Eligible patients were required to be on regular transfusion schedules and to have complete endocrine evaluations performed both at study entry and at the end of the follow-up period. Patients who were pregnant during the study period, had endocrine disorders unrelated to thalassemia, used exogenous hormones that could interfere with endocrine assessment, had acute systemic illness, or had previously undergone hematopoietic stem cell transplantation were excluded from the analysis. For comparative evaluation of endocrine parameters, an age-matched control group of healthy women without a history of hematological disease was included. A total of 15 adult women with transfusion-dependent β-thalassemia were included in the study, along with an age-matched healthy control group consisting of 22 women.

### 2.2. Clinical and Laboratory Assessments

Demographic and clinical data, including age, marital status, transfusion history, splenectomy status, and reproductive history (primary or secondary amenorrhea or spontaneous menstruation), were obtained from medical records. Bone mineral density was assessed and categorized as normal, osteopenia, or osteoporosis according to standard criteria. Routine laboratory data included hemoglobin concentration, serum ferritin, serum iron, total iron-binding capacity, vitamin B12, folate, and 25-hydroxyvitamin D.

Endocrine assessments were performed at baseline and at the two-year follow-up. Morning fasting venous blood samples were collected for measurement of AMH, follicle-stimulating hormone (FSH), luteinizing hormone (LH), estradiol (E2), thyroid-stimulating hormone (TSH), and prolactin (PRL). In women with amenorrhea, hormonal measurements were performed on morning fasting samples without regard to cycle timing, as no defined follicular phase was present.

### 2.3. Statistical Analysis

Descriptive statistics were summarized as mean, standard deviation, median, and range. The Shapiro–Wilk test was used to assess the normality of data distribution. Within-patient comparisons between baseline and the two-year follow-up were performed using paired *t*-tests for normally distributed variables and the Wilcoxon signed-rank test for non-normally distributed variables. Comparisons between the thalassemia group and healthy controls were conducted using independent *t*-tests for normally distributed data and the Mann–Whitney U test for variables that did not meet normality assumptions. A two-sided *p*-value of < 0.05 was considered statistically significant. Given the sample size, analyses were exploratory in nature. All statistical analyses were performed using IBM SPSS Statistics for Windows, version 31.0 (IBM Corp., Armonk, NY, USA).

### 2.4. Ethical Approval

The study protocol was approved by the Ethics Committee of Istanbul Medeniyet University Göztepe City Hospital (Clinical Research Ethics Committee) (Approval No. 2021/0327). Written informed consent was obtained from all participants, and the study was conducted in accordance with the principles of the Declaration of Helsinki.

## 3. Results

A total of 15 adult women with TDT were included in the analysis. The median age of the cohort was 27 years. Patients had received a median of 459 transfusions. Cardiac magnetic resonance imaging T2* (MRI T2*) values demonstrated preserved myocardial iron status, with a median value of 23.5 ms, whereas liver MRI T2* values indicated mild-to-moderate hepatic iron overload, with a median value of 4.5 ms. Serum ferritin levels showed marked interindividual variability, ranging widely across patients (241–18,983 ng/mL).

Baseline biochemical assessment revealed frequent metabolic and micronutrient abnormalities within the TDT cohort. The median serum vitamin D level was 15.7 ng/mL, consistent with vitamin D insufficiency. Median folate and vitamin B12 levels were 9.1 ng/mL and 354 pg/mL, respectively. Routine iron parameters demonstrated elevated serum iron and reduced total iron-binding capacity, reflecting chronic transfusion-related iron exposure. Thyroid function parameters and PRL levels were generally within reference ranges.

Ten patients (66.7%) had previously undergone splenectomy. Primary amenorrhea was observed in two patients (13.3%), whereas secondary amenorrhea was present in six patients (40%). Spontaneous menstruation was reported by thirteen women (86.7%). Six patients (40%) were married, and three (20%) had at least one child. Bone mineral density assessment demonstrated normal findings in three patients (20%), osteopenia in five patients (33.3%), and osteoporosis in seven patients (46.7%).

Baseline endocrine hormone levels demonstrated substantial interindividual variability. Median baseline values were 1.37 µIU/mL for TSH, 2.31 IU/L for LH, 4.11 IU/L for FSH, 43 pg/mL for E2, and 2.81 ng/mL for AMH.

At the two-year follow-up, median hormone levels remained largely unchanged, with values of 1.69 µIU/mL for TSH, 3.94 IU/L for LH, 4.4 IU/L for FSH, 39 pg/mL for E2, and 2.2 ng/mL for AMH. Within-patient comparisons using the Wilcoxon signed-rank test revealed no statistically significant temporal changes in any of the assessed endocrine parameters over the two-year follow-up period (Table 1). AMH levels showed pronounced interindividual variability at both time points.

Endocrine parameters were also compared between women with TDT and an age-matched healthy control group (n = 22). Median hormone levels in the control group were 1.525 µIU/mL for TSH, 3.48 IU/L for LH, 5.75 IU/L for FSH, 37.45 pg/mL for E2, and 1.81 ng/mL for AMH. Comparisons between the TDT and control groups using the Mann–Whitney U test demonstrated no statistically significant differences for any endocrine parameter (Table 2).

Despite the absence of statistically significant differences in hormone levels over time or between groups, hypogonadism and menstrual disturbances remained clinically prevalent within the TDT cohort. Overall endocrine profiles remained stable during the follow-up period, and no newly diagnosed overt endocrine disorders were identified.

## 4. Discussion

Endocrine complications remain a major determinant of long-term morbidity in TDT, particularly as survival into adulthood has increasingly improved [9]. Among endocrine sequelae, hypogonadism represents the most frequently reported abnormality and remains a central clinical concern. Iron-related injury to the hypothalamic–pituitary–gonadal (HPG) axis is known to develop predominantly during childhood and adolescence and may remain clinically silent for prolonged periods before overt dysfunction becomes apparent in adulthood [10,11].

In this retrospective longitudinal study, endocrine and iron overload parameters were evaluated over a two-year follow-up period in 15 adult women with TDT to examine the adult manifestations of iron-related HPG axis involvement. In line with previous reports, hypogonadism was clinically prevalent in our cohort [11]. However, only a limited association was observed between endocrine parameters and contemporaneous serum ferritin levels or cardiac and hepatic MRI T2* measurements. These findings suggest that gonadal dysfunction identified in adulthood reflects cumulative endocrine injury acquired earlier in life, which may persist and be only partially modifiable by iron control achieved later in the disease course [12].

Pituitary iron deposition, particularly affecting gonadotroph cells, plays a central role in the pathogenesis of hypogonadism. The ability to detect pituitary iron accumulation by imaging before the development of clinically overt endocrine failure further supports the concept of early and subclinical central involvement [11,13]. Nevertheless, the findings of the present study do not support a uniform pattern of hypogonadism.

Despite the absence of marked elevations in gonadotropin levels, more than half of the patients in our cohort experienced amenorrhea, suggesting a heterogeneous pattern of hypogonadism in adult women with TDT that differs from the classical model of hypergonadotropic ovarian failure. This dissociation between clinical and biochemical findings points toward a more complex heterogeneous pathophysiology.

In this context, the pronounced variability in AMH observed over the two-year follow-up emerged as a sensitive indicator of subclinical and interindividual differences in ovarian reserve, even in the presence of stable gonadotropin levels. Taken together, these findings indicate that hypogonadism in this patient population cannot be explained by a single fixed central or peripheral mechanism. Rather, it appears to represent a spectrum of dysfunction involving early-established and stabilized central impairment combined with variable degrees of gonadal contribution [14].

The stability of FSH, LH, as well as TSH and PRL levels over the two-year follow-up, together with their comparability to those of healthy controls, suggests that no overt progressive deterioration of hypothalamic–pituitary function occurred during this period. Preservation of thyroid and PRL axes further implies that different endocrine axes may exhibit varying susceptibility to iron burden and temporal exposure in TDT [12].

Adequate contemporary iron control may represent an important factor contributing to the stability of endocrine parameters observed in our cohort. Longitudinal studies have demonstrated that regular and effective chelation reduces the risk of developing new endocrinopathies and may limit the progression of existing dysfunction [15]. Nevertheless, even in well-chelated adult patients with satisfactory systemic iron indices, continued endocrine surveillance remains clinically important.

Bone health constitutes another major dimension of endocrine morbidity in TDT. In our cohort, only 20% of patients exhibited normal bone mineral density, while the majority had osteopenia or osteoporosis. This finding is consistent with previous reports and reflects the multifactorial impact of hypogonadism, chronic anemia, iron toxicity, vitamin D deficiency, and altered bone metabolism [16,17]. These observations underscore that endocrine complications in TDT extend beyond isolated gonadal abnormalities and require comprehensive, multidisciplinary evaluation.

Normal gonadotropin and pituitary hormone levels should not be interpreted as an absence of endocrine risk in adult women with TDT. Similarly, satisfactory control of systemic iron indices alone is insufficient to ensure preserved reproductive function. The frequent occurrence of menstrual irregularities despite normal biochemical parameters highlights the importance of incorporating clinical history and menstrual patterns into routine endocrine assessment. Therefore, follow-up of adult women with TDT should adopt a holistic approach that integrates clinical phenotype, ovarian reserve markers, and laboratory data, a strategy that is particularly critical for timely and appropriate fertility counseling and reproductive planning.

Taken together, these findings suggest that reproductive endocrine dysfunction in adult women with TDT reflects a heterogeneous and potentially cumulative process that may persist despite apparently stable biochemical parameters and adequate iron control. The findings should be interpreted in light of the retrospective design and modest sample size, which are inherent to studies of rare adult TDT cohorts. While the two-year follow-up provides insight into short-term endocrine trajectories, longer observation periods would be valuable to better characterize slowly evolving or late-onset dysfunction. In addition, the absence of dynamic endocrine testing limits detailed mechanistic interpretation of hypogonadism. Nonetheless, the observed dissociation between biochemical stability and persistent clinical hypogonadism underscores the complexity of gonadal axis injury in adult TDT patients and supports the need for longitudinal, phenotype-oriented endocrine surveillance beyond reliance on single-time-point laboratory measures.

In this context, the present findings emphasize that apparent biochemical stability does not necessarily equate to preserved reproductive endocrine health in adult women with TDT. Rather than reflecting reversible or transient dysfunction, persistent hypogonadism may represent cumulative endocrine injury with variable clinical expression, underscoring the importance of continued, individualized endocrine follow-up across adulthood.

### Strengths and Limitations

The strengths of this study include its longitudinal design, comprehensive endocrine and biochemical profiling, and focus on adult women with transfusion-dependent β-thalassemia receiving regular transfusion and chelation therapy. Additionally, the inclusion of an age-matched healthy control group allows contextual interpretation of endocrine parameters.

Several limitations should be acknowledged. The relatively small sample size limits statistical power and precludes definitive conclusions. Furthermore, the exploratory nature of the analyses may reduce the ability to detect subtle differences over time or between groups. Nevertheless, the findings provide valuable insight into endocrine stability and variability in a clinically relevant patient population and may serve as a foundation for future larger-scale studies.

## 5. Conclusions

Hypogonadism remains a frequent endocrine complication in adult women with transfusion-dependent β-thalassemia, even in the context of contemporary iron chelation. Over a two-year follow-up period, reproductive endocrine parameters remained largely stable, supporting the concept of persistent rather than progressive endocrine injury acquired earlier in life. Variability in anti-Müllerian hormone levels may reflect subtle or subclinical impairment of ovarian reserve, despite preserved gonadotropin secretion. Collectively, these findings highlight the heterogeneous nature of gonadal axis dysfunction in this population and support the value of longitudinal, phenotype-oriented endocrine surveillance, including systematic assessment of gonadal hormones, ovarian reserve markers, thyroid function, and iron-related parameters.

## Figures and Tables

**Table 1:** Endocrine measurements at baseline and 2-year follow-up in women with transfusion-dependent β-thalassemia (n = 15).

Parameter	Baseline, Median (IQR)	2-Year Follow-Up, Median (IQR)	*p*-Value *
TSH (µIU/mL)	1.37 (0.84–1.94)	1.69 (1.30–2.30)	0.594
LH (IU/L)	2.31 (1.27–6.18)	3.94 (1.50–6.60)	0.173
FSH (IU/L)	4.11 (2.32–5.43)	4.4 (1.70–6.60)	0.152
E2 (pg/mL)	43 (25–71)	39 (15–83)	0.861
AMH (ng/mL)	2.81 (1.68–3.79)	2.2 (1.67–3.29)	0.307

Legend: Values are presented as median (interquartile range). * Within-patient comparisons were performed using the Wilcoxon signed-rank test. Abbreviations: TSH, thyroid-stimulating hormone; LH, luteinizing hormone; FSH, follicle-stimulating hormone; E2, estradiol; AMH, anti-Müllerian hormone.

**Table 2:** Comparison of endocrine measurements between women with transfusion-dependent β-thalassemia and healthy controls.

Parameter	TDT (n = 15), Median (IQR)	Controls (n = 22), Median (IQR)	*p*-Value ^†^
TSH (µIU/mL)	1.69 (1.30–2.30)	1.525 (1.04–1.99)	0.346
LH (IU/L)	3.94 (1.50–6.60)	3.48 (2.35–5.79)	0.804
FSH (IU/L)	4.4 (1.70–6.60)	5.75 (4.91–7.28)	0.094
E2 (pg/mL)	39 (15–83)	37.45 (28.90–52.67)	0.700
AMH (ng/mL)	2.2 (1.67–3.29)	1.80 (1.50–2.93)	0.497

Legend: Values are presented as median (interquartile range). ^†^ Group comparisons were performed using the Mann–Whitney U test. Abbreviations: TSH, thyroid-stimulating hormone; LH, luteinizing hormone; FSH, follicle-stimulating hormone; E2, estradiol; AMH, anti-Müllerian hormone.

## Data Availability

The data supporting the findings of this study are not publicly available due to ethical and privacy restrictions, but are available from the corresponding author upon reasonable request, subject to approval by the institutional ethics committee.

## Abbreviations

The following abbreviations are used in this manuscript:

BTM	βeta-Thalassemia Major
TDT	Transfusion-Dependent Β-Thalassemia
MR T2*	Magnetic Resonance Imaging T2* Measurements
AMH	Anti-Müllerian Hormone
TSH	Thyroid-Stimulating Hormone
LH	Luteinizing Hormone
FSH	Follicle-Stimulating Hormone
E2	Estradiol
